# The therapeutic validity and effectiveness of physiotherapeutic exercise following total hip arthroplasty for osteoarthritis: A systematic review

**DOI:** 10.1371/journal.pone.0194517

**Published:** 2018-03-16

**Authors:** Annet Wijnen, Sjoukje E. Bouma, Gesine H. Seeber, Lucas H. V. van der Woude, Sjoerd K. Bulstra, Djordje Lazovic, Martin Stevens, Inge van den Akker-Scheek

**Affiliations:** 1 University Hospital of Orthopedics and Trauma Surgery Pius-Hospital, Medical Campus University of Oldenburg, Oldenburg, Germany; 2 Department of Orthopedics, University of Groningen, University Medical Center Groningen, Groningen, The Netherlands; 3 University of Groningen, University Medical Center Groningen, Center for Human Movement Sciences, Groningen, The Netherlands; University of Michigan, UNITED STATES

## Abstract

**Objective:**

To assess the therapeutic validity and effectiveness of physiotherapeutic exercise interventions following total hip arthroplasty (THA) for osteoarthritis.

**Data sources:**

The databases Embase, MEDLINE, Cochrane Library, CINAHL and AMED were searched from inception up to February 2017.

**Eligibility criteria:**

Articles reporting results of randomized controlled trials in which physiotherapeutic exercise was compared with usual care or with a different type of physiotherapeutic exercise were included, with the applied interventions starting within six months after THA. Only articles written in English, German or Dutch were included.

**Study appraisal:**

Therapeutic validity (using the CONTENT scale) and risk of bias (using both the PEDro scale and the Cochrane Collaboration’s tool) were assessed by two researchers independently. Characteristics of the physiotherapeutic exercise interventions and results about joint and muscle function, functional performance and self-reported outcomes were extracted.

**Results:**

Of the 1124 unique records retrieved, twenty articles were included. Only one article was considered to be of high therapeutic validity. Description and adequacy of patient selection were the least reported items. The majority of the articles was considered as having potentially high risk of bias, according to both assessment tools. The level of therapeutic validity did not correspond with the risk of bias scores. Because of the wide variety in characteristics of the physiotherapeutic exercise and control interventions, follow-up length and outcome measures, limited evidence was found on the effectiveness of physiotherapeutic exercise following THA.

**Conclusion:**

The insufficient therapeutic validity and potentially high risk of bias in studies involving physiotherapeutic exercise interventions limit the ability to assess the effectiveness of these interventions following THA. Researchers are advised to take both quality scores into account when developing and reporting studies involving physiotherapeutic exercise. Uniformity in intervention characteristics and outcome measures is necessary to enhance the comparability of clinical outcomes between trials.

## Introduction

Osteoarthritis (OA) is a common degenerative joint disorder, the hip being one of the most affected joints [1]. Predominant symptoms include pain, stiffness, instability, swelling and muscle weakness, leading to loss of function, disability and reduced quality of life [1–3]. Major risk factors for developing OA are older age and female gender [1]. Obesity, previous joint injury, muscle weakness and genetic factors are also identified as risk factors for developing OA [2]. Treatment of OA may be pharmacological, non-pharmacological or surgical, with total hip arthroplasty (THA) as the most frequently performed surgical procedure for end-stage hip OA [4]. Because of an aging population and the increasing prevalence of obesity, the incidence of hip OA is rising in the Western world, resulting in a higher demand for THA [3,5]. In the Netherlands, the yearly number of primary THAs increased from 23,330 to 28,798 between 2010 and 2015 [6]. In 2015, 86.7% of THAs were performed because of OA. In Germany, 227,293 primary THAs were performed in 2015 [7]. The annual number of THAs is expected to further increase in the coming years [5,8].

**Fig 1:**
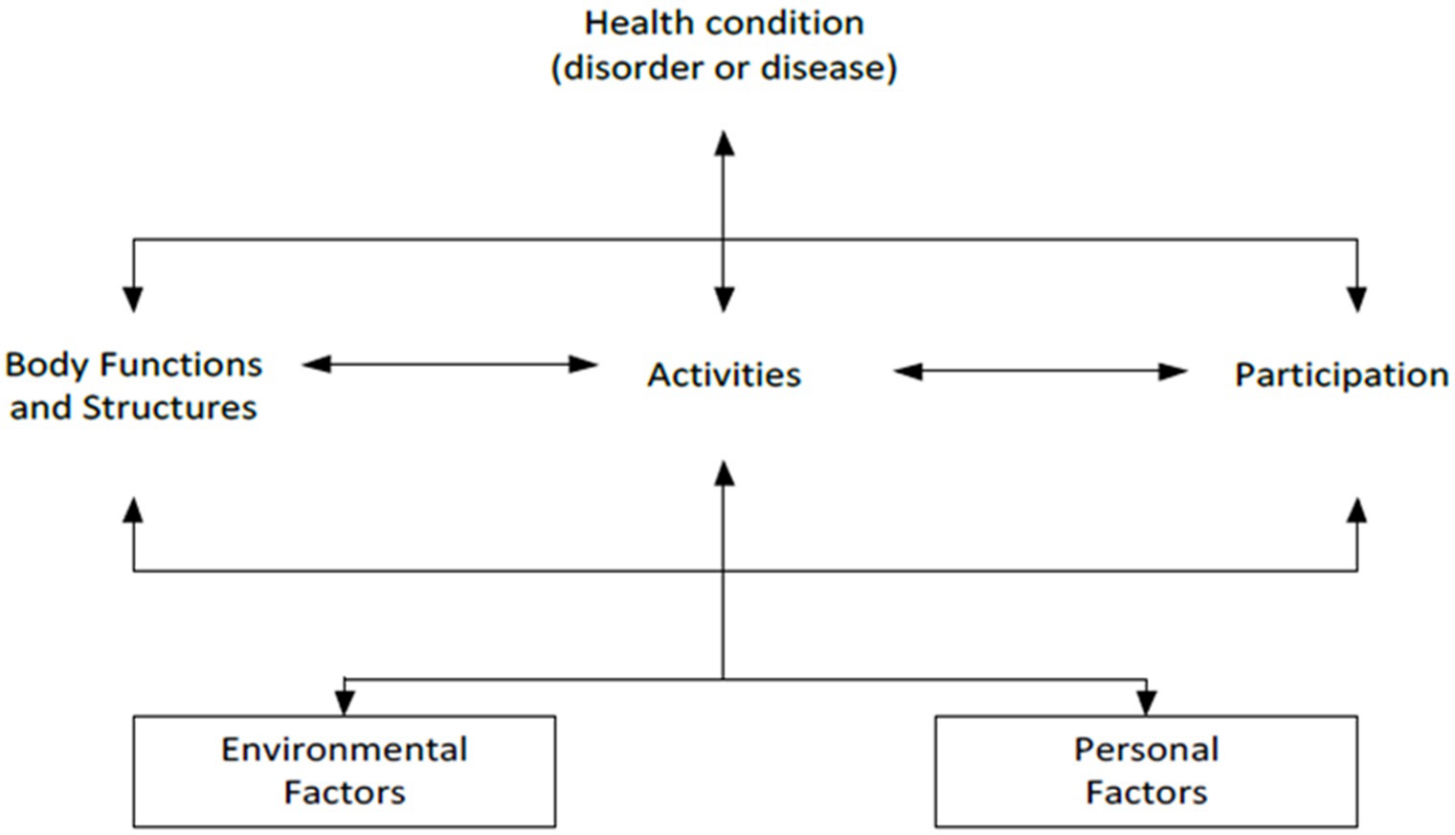
The International Classification of Function, Disability and Health (ICF) model [13].

As part of the rehabilitation program following THA, patients are usually administered physiotherapeutic exercise [9,10]. Physiotherapeutic exercise can be defined as any movement intervention, such as joint-specific exercises for improving strength and range of motion, stretching, proprioceptive exercises and general aerobic conditioning, that is performed in order to improve physical health and restore normal function [11,12]. According to the International Classification of Functioning, Disability and Health (ICF) model (Fig 1), the functioning of an individual can be described at three levels: body functions and structures, activities and participation. Functioning is also the result of the interaction between an individual’s health condition and contextual (environmental and personal) factors [13]. It is known that patients may still have deficits in body functions and structures (muscle strength and postural stability) and activities (decreased walking speed) up to two years after THA [14–16]. Better insight into the effectiveness of physiotherapeutic exercise following THA is therefore required.

Physiotherapeutic exercise can be considered as a complex intervention, as it can be administered in very different ways and settings. As a result, heterogeneity of effect may occur [17]. Herbert and Bø therefore recommend that systematic reviews should explicitly examine the therapeutic validity of these interventions [17]. To this end, a nine-item rating scale for the therapeutic validity of therapeutic exercise was developed in a four-round Delphi study named the Consensus on Therapeutic Exercise Training (CONTENT) scale [18]. Therapeutic validity was defined as the potential effectiveness of a specific intervention given to a potential target group of patients. So far, the CONTENT scale has been used in three systematic reviews [18–20].

Several systematic reviews have previously been conducted on the effectiveness of physiotherapeutic exercise following THA [21–23]. These reviews demonstrate that there is insufficient evidence to give a definite answer due to the potentially high risk of bias in many trials, the diversity in trial interventions and the generally small sample sizes. For this reason, it is not yet possible to create an evidence-based protocol for type and timing of physiotherapeutic exercise after THA. However, none of these reviews has systematically evaluated the content and therapeutic validity of the applied interventions as recommended [17,18].

The aim of our study was therefore to assess therapeutic validity in addition to effectiveness of physiotherapeutic exercise interventions in patients following THA for OA. To examine therapeutic validity and effectiveness of physiotherapeutic exercise, we reviewed randomized controlled trials (RCTs) comparing a physiotherapeutic exercise intervention with usual care or a different physiotherapeutic exercise intervention in patients who underwent unilateral primary THA because of OA. We hypothesized that studies of high therapeutic validity could demonstrate a positive effect on joint and muscle function, functional performance and self-reported outcomes in patients after THA.

## Methods

This systematic review was conducted following the Preferred Reporting Items for Systematic reviews and Meta-Analyses (PRISMA) guidelines (S1 File).

### Data sources and searches

The following five databases were searched for relevant articles from inception up to 24 February 2017: Embase, MEDLINE, Cochrane Library, CINAHL and AMED. The search strategies for the different databases, which were optimized by a librarian, are shown in S2 File. The reference lists of the included articles were manually searched for additional relevant references.

### Study selection

Articles describing the results of RCTs comparing postoperative physiotherapeutic exercise with usual care or comparing two different postoperative physiotherapeutic exercise interventions were included in this review. Articles were also included if the described trial met the following eligibility criteria: (1) the study included only patients who underwent unilateral primary THA (replacement of head and socket) because of OA; (2) the start of the intervention was within six months after THA; (3) the applied intervention consisted of land-based or water-based physiotherapeutic exercise (in both the inpatient and outpatient setting); (4) pre- and post-intervention measurements were conducted for both study groups; (5) outcomes from at least one of the following categories were reported: joint and muscle function (corresponding with body functions from the ICF model, e.g. strength, range of motion (ROM)), functional performance (corresponding with activities from the ICF model, e.g. walking speed, stair-climbing performance) and self-reported outcomes (e.g. questionnaires evaluating quality of life or pain); and (6) the article was written in English, German or Dutch. Articles reporting trials that included patients who were undergoing partial hip replacement (head or socket), hip resurfacing or revision surgery were excluded. Other physiotherapeutic modalities such as manual therapy, osteopathy and electric stimulation therapy were not considered as physiotherapeutic exercise therapy according to the definition used in this study. Hence articles reporting trials in which these modalities were applied as intervention were excluded. Lastly, articles reporting the effect on outcome measures for the acute postoperative phase following THA (e.g. length of hospital stay, wound leakage) or for specific muscle properties (e.g. thickness, morphology, architecture) were not included in this systematic review.

Two researchers (AW and SEB) independently assessed the eligibility of all identified articles. The first selection was made based on title, abstract and language. The full text of the remaining articles was subsequently retrieved to assess whether the article met the predefined inclusion criteria. Disagreements between the two researchers were solved in a consensus meeting.

### Data extraction and quality assessment

The following data were extracted from each included article by SEB: country and year of publication, sample size, participant characteristics (age and gender), characteristics of the physiotherapeutic exercise intervention (type, setting, supervision, duration, frequency, intensity, start and length of follow-up), characteristics of the control intervention (frequency and intensity) and main results in each outcome category (joint and muscle function, functional performance and self-reported outcomes). Type of physiotherapeutic exercise was divided into the following three categories: strengthening exercise (explicitly aimed at improving muscle strength and using external resistance), aerobic exercise and functional exercise (focused on training functional tasks, but not explicitly on improving muscle strength or endurance).

The therapeutic validity of the physiotherapeutic exercise interventions was assessed by two researchers independently (SEB and GHS) using the CONTENT scale (S3 File). The CONTENT scale consists of five domains (patient eligibility, competences and setting, rationale, content, and adherence), with nine items in total. Each item is rated as “yes” (1 point) or “no” (0 points). The scores on the nine items are summed to calculate a total score. Studies with at least 6 points are considered as being of high therapeutic validity [18]. Disagreements between the researchers in assessing therapeutic validity were solved in a consensus meeting.

Risk of bias of the studies was assessed by two researchers independently (AW and SEB) using both the Physiotherapy Evidence Database (PEDro) scale and the Cochrane Collaboration’s tool. It was chosen to judge risk of bias twice to assess whether there are differences in the final judgment according to these assessment tools. The PEDro scale is an 11-item scale, which is found to be a reliable tool for use in systematic reviews of RCTs evaluating physiotherapeutic interventions [24]. Each item scoring “yes” contributes 1 point to the total score, except for the first item, which relates to external validity. The total PEDro score thus ranges from 0 to 10 points. Studies with a total score of at least 6 points are considered to be of adequate quality [24,25]. The Cochrane Collaboration’s tool consists of six domains (selection bias, performance bias, detection bias, attrition bias, reporting bias and other bias), with seven items to be scored in total [26]. Each item is rated as “low risk”, “high risk” or “unclear risk” of bias. Studies were considered to be of adequate quality when the items’ random sequence generation, allocation concealment and blinding of outcome assessment were rated as low risk of bias. This method of defining adequate quality has been previously used in assessing risk of bias in physiotherapeutic exercise trials [25]. Disagreements between the researchers in assessing risk of bias were solved in a consensus meeting. A third researcher (MS) was consulted to give a final judgment when disagreement persisted.

### Data synthesis

Due to the heterogeneity of outcome measures and reported units of measurement, meta-analyses of data were not considered appropriate. Therefore, a narrative synthesis of data was used to evaluate the included studies and to give recommendations for future research. Furthermore, it was qualitatively assessed whether there seemed to be a match between the results of therapeutic validity assessments of physiotherapeutic exercise interventions and the risk of bias scores of the included studies.

## Results

### Study selection

**Fig 2:**
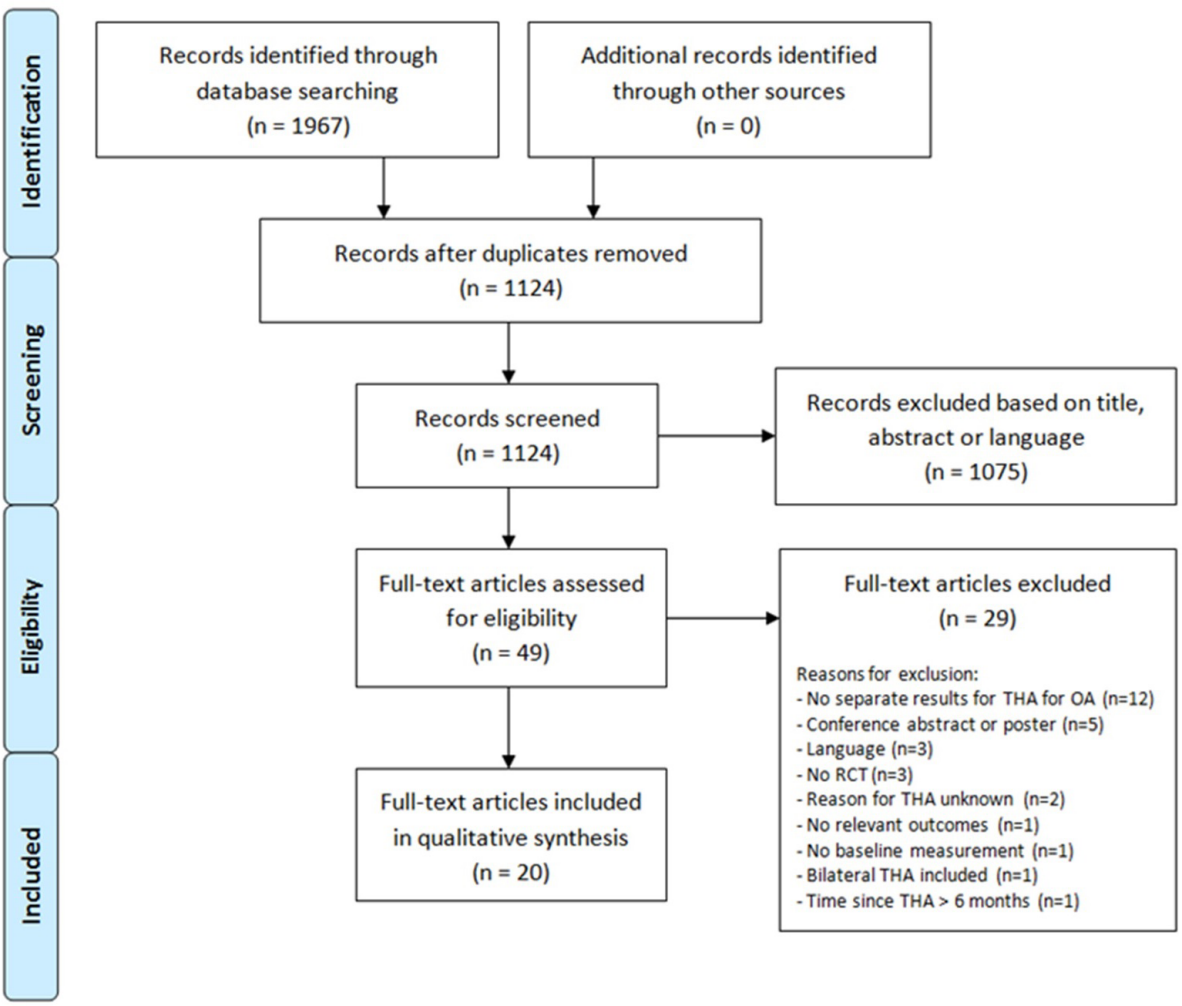
Flow diagram of the article selection process. THA: total hip arthroplasty; OA: osteoarthritis; RCT: randomized controlled trial.

The aforementioned search strategy identified 1967 records, which contained 843 duplicates. Of the 1124 unique records, 1075 articles were excluded based on title, abstract or language. Twenty of the remaining 49 potentially relevant articles met the inclusion criteria and were therefore included in the systematic review. The list of excluded full-text articles with the reasons for exclusion is presented in S4 File. Manually searching the reference lists of included articles did not lead to finding additional articles. A flow diagram of the selection process is shown in Fig 2.

### Study characteristics

The characteristics of the twenty included articles are shown in Table 1. Seven articles were found that investigated the effect of strengthening exercises [27–33]. Two articles focused on the effect of aerobic exercise [34,35]. The remaining eleven articles investigated the effect of functional exercise [36–46]. In three of the articles involving functional exercise, early full weight-bearing was instructed in the intervention group (IG), while the control group (CG) followed a partial weight-bearing regime (until three months after THA) [37,44,45]. The start of the interventions varied from one day to three months postoperatively. The follow-up periods ranged from fifteen days to five years postoperatively. Duration of the interventions varied between two and twelve weeks. Self-reported outcomes were assessed in eighteen articles (90%). Fourteen articles (70%) reported outcomes of joint and muscle function, and eleven articles (55%) investigated the effect of the intervention on functional performance.

**Table 1:** Study characteristics of the included articles.

				Physiotherapeutic exercise intervention					Control intervention			Results^f^		
Author (year, location)	Study groups (n)	Age (years)^a^	Women (n (%))	Type	Setting^b^ (supervision^c^)	Duration (frequency^d^)	Intensity^e^	Start and follow-up^d^	Description	Frequency^d^	Intensity^e^	Joint & muscle function	Functional performance	Self-reported outcomes
Husby (2009, Norway)	IG: 12; CG: 12	IG: 58 ± 5; CG: 56 ± 8	15 (63)	Strengthening (+ control intervention)	I; L (Y)	4w (5x pw)	4 series of 5RM	1w; 5w	Usual care	5x pw	1h	↑ / =	N/A	=
Husby (2010, Norway)	IG: 12; CG: 12	IG: 58 ± 5; CG: 56 ± 8	15 (63)	Strengthening (+ control intervention)	I; L (Y)	4w (5x pw)	4 series of 5RM	1w; 12m	Usual care	5x pw	1h	↑ / =	N/A	=
Mikkelsen (2012, Denmark)	IG: 23; CG: 21	67.3 ± 7	16 (36)	Strengthening	O; L (N)	12w (7x pw, 2x day)	1x10 reps	1d; 12w	Usual care	7x pw, 2x day	1x10 reps	=	=	=
Mikkelsen (2014, Denmark)	IG: 32; CG: 30	IG: 64.8 ± 8; CG: 65.1 ± 10	26 (42)	Strengthening	O; L (P)	10w (2x pw; 5x pw home-based exercise)	30–40 min	<1w; 10w-12m	Usual care	7x pw, 2x day	1x10 reps	=	↑ / =	=
Nankaku (2016, Japan)	IG: 14; CG: 14	IG: 60.5 ± 6.4; CG: 60.8 ± 7.5	24 (86)	Strengthening (+ control intervention)	I; L (Y)	4w (5x pw)	3x8-12 reps	NR; 4w	Usual care	NR	NR	↑ / =	↑	↑
Okoro (2016, UK)	IG: 25; CG: 24	IG: 65.2 ± 9.1; CG: 66.3 ± 11.0	25 (51)	Strengthening	O; L (P)	6w (5x pw)	Range of reps	4-7d; 9-12m	Usual care	NR	NR	=	= / ↓	N/A
Suetta (2004, Denmark)^g^	IG: 13; CG: 12	IG: 69 (range: 60–86); CG: 68 (range: 62–78)	19 (53)	Strengthening (+ control intervention)	O; L (Y)	12w (3x pw)	Progressively increased from 20RM to 8RM	±7d; 12w	Usual care	7x pw, 2x day	1h	↑	↑ / =	N/A
Maire (2004, France)	IG: 7; CG: 7	75.1 ± 4.8	12 (86)	Aerobic (+ control intervention)	I; L (NR)	6w (3x pw)	30 min	1w; 2m	Usual care	7x pw	2h	N/A	N/A	=
Maire (2006, France)	IG: 7; CG: 7	75.1 ± 4.8	12 (86)	Aerobic (+ control intervention)	I; L (NR)	6w (3x pw)	30 min	1w; 12m	Usual care	7x pw	2h	N/A	N/A	↑
Beaupre (2014, Canada)	IG: 11; CG: 10	53.4 ± 9.3	10 (48)	Functional	O; L/W (NR)	3m (2x pw)	2.5h	6w; 12m	Usual care	NR	NR	=	=	=
Galea (2008, Australia)	IG: 11; CG: 12	IG: 68.6 ± 9.7; CG: 66.6 ± 7.9	16 (70)	Functional	I; L (Y)	8w (2x pw)	45 min	8w; 16w	Unsupervised home-based exercise	NR	NR	N/A	=	=
Giaquinto (2010, Italy)	IG: 31; CG: 39	IG: 70.1 ± 8.5; CG: 70.6 ± 8.4	IG: 21 (68); CG: 26 (67)	Functional	I; W (NR)	3w (6x pw)	40 min	<10d; 6m	Land-based therapy	6x pw	40 min	N/A	N/A	↑
Heiberg (2012, Norway)	IG: 35; CG: 33	66 (95% CI: 64, 67)	35 (51)	Functional	O; L (Y)	6w (2x pw)	70 min	3m; 12m	No supervised physiotherapy	NR	NR	=	↑ / =	=
Heiberg (2016, Norway)	IG: 30; CG: 30	70 (95% CI: 68, 72)	34 (57)	Functional	O; L (Y)	6w (2x pw)	70 min	3m; 5y	No supervised physiotherapy	NR	NR	=	=	=
Johnsson (1988, Sweden)	IG: 14; CG: 16	IG: 70 (range: 58–67); CG: 66 (range: 50–74)	13 (43)	Functional	O; L (NR)	2-3m (0,5-2x pw)	45 min	2m; 6m	No organized physiotherapy	NR	NR	=	=	=
Monaghan (2016, Ireland)	IG: 32; CG: 31	IG: 68 ± 8; CG: 69 ± 9	20 (32)	Functional (+ control intervention)	O; L (Y)	6w (2x pw)	35 min	12w; 18w	Usual care	NR	NR	=	↑	↑/ =
Umpierres (2014, Brazil)	IG: 54; CG: 52	61.4 ± 15.0	57 (54)	Functional	I/O; L (P)	2w (NR)	NR	1d; 15d	No supervised physiotherapy	NR	NR	↑ / =	N/A	=
Bodén (2004, Sweden)	IG: 10; CG: 10	IG: 54 (range: 44–59); CG: 55 (range: 44–63)	11 (55)	Functional and early full weight-bearing	O; L (N)	NR	NR	NR; 24m	Partial weight-bearing and home-based exercise	NR	NR	N/A	N/A	=
Monticone (2014, Italy)	IG: 50; CG: 50	69 ± 8	60 (60)	Functional and early full weight-bearing	I; L (Y)	3w (5x pw)	90 min	4-7d; 12m	Partial weight-bearing and center-based exercise	5x pw	90 min	N/A	N/A	↑
Ström (2006, Sweden)	IG: 17; CG: 19	54.4 (range: 26–63)	19 (53)	Functional and early full weight-bearing	O; L/W (Y)	3m (NR)	NR	NR; 12m	Partial weight-bearing and a self-training program	NR	NR	=	N/A	N/A

CG: control group; CI: confidence interval; IG: intervention group; NR: not reported.

^a^ Age is shown as mean +/- SD, unless stated otherwise.

^b^ Intervention: inpatient (I) or outpatient (O); land-based (L) or water-based (W).

^c^ Whether the intervention was supervised: yes (Y), no (N) or partially (P).

^d^ Shown as time since total hip arthroplasty: days (d), weeks (w), months (m) or years (y); pw: per week.

^e^ Minutes (min); hours (h); repetition maximum (RM); repetitions (reps).

^f^ Effect of the intervention: ↑ significant difference in favor of IG; = no significant difference between IG and CG; ↓ significant difference in favor of CG; N/A: not applicable as the article did not include this category of outcome measures.

^g^ In this study a second intervention group was included that received neuromuscular electrical stimulation (NMES) in addition to the standard rehabilitation program (n = 11). However, the characteristics and results of this intervention group are not presented, since NMES was defined as an exclusion criterion for this systematic review.

### Therapeutic validity

The assessment of the therapeutic validity using the CONTENT scale is shown in Table 2. Absolute agreement was achieved for 149 out of 180 items (82.7%). All disagreements were solved without the need to consult a third assessor. The median score of the therapeutic validity was 2 (range 0–7). Only one article was considered to be of high therapeutic validity (defined as a score ≥ 6). Monitoring was the most reported item: half of the articles gave a description of the monitoring of the therapy progression. In eight articles (40%) *a priori* intentions and hypotheses were described, while a rationale for the specific content and intensity of the therapeutic exercise was given in five articles (25%). The items description of patient eligibility and adequate patient eligibility achieved the lowest total scores. These items were reported in two articles (10%). It appears that the articles, which focus on strengthening exercises had higher scores on therapeutic validity than those involving other physiotherapeutic exercise interventions. In general, articles focusing on strengthening exercises seemed to score higher on the items setting and therapist, intensity, monitoring, and adherence.

**Table 2:** Results of the therapeutic validity assessment using the CONTENT scale.

Study	Patient eligibility		Setting & therapist	Rationale		Content			Adherence	Total score
	Described	Adequate		Study	Intervention	Intensity	Monitored	Personalized		
*Strengthening exercise*										
Husby (2009)	No	No	Yes	Yes	Yes	Yes	Yes	No	No	5
Husby (2010)	No	No	Yes	Yes	Yes	Yes	Yes	No	No	5
Mikkelsen (2012)	No	No	No	Yes	Yes	Yes	No	Yes	Yes	5
Mikkelsen (2014)	Yes	Yes	Yes	Yes	No	Yes	Yes	No	Yes	7
Nankaku (2016)	No	No	Yes	No	No	No	No	No	Yes	2
Okoro (2016)	Yes	No	Yes	No	No	No	Yes	No	Yes	4
Suetta (2004)	No	No	Yes	No	No	No	Yes	No	No	2
*Aerobic exercise*										
Maire (2004)	No	No	No	No	No	No	Yes	Yes	No	2
Maire (2006)	No	No	No	Yes	Yes	Yes	Yes	No	No	4
*Functional exercise*										
Beaupre (2014)	No	No	No	No	No	No	No	No	No	0
Galea (2008)	No	No	No	Yes	No	No	No	No	Yes	2
Giaquinto (2010)	No	No	No	No	No	No	No	No	No	0
Heiberg (2012)	No	No	No	No	No	No	Yes	Yes	No	2
Heiberg (2016)	No	No	No	No	No	No	Yes	No	No	1
Johnsson (1988)	No	No	No	No	No	No	No	No	No	0
Monaghan (2016)	No	No	Yes	No	Yes	No	Yes	No	Yes	4
Umpierres (2014)	No	No	Yes	Yes	No	No	No	No	No	2
*Functional exercise and early full weight-bearing*										
Bodén (2004)	No	No	No	No	No	No	No	No	No	0
Monticone (2014)	No	Yes	No	Yes	No	No	No	No	No	2
Ström (2006)	No	No	No	No	No	No	No	No	No	0
Total score	2 (10%)	2 (10%)	8 (40%)	8 (40%)	5 (25%)	5 (25%)	10 (50%)	3 (15%)	6 (30%)	

### Risk of bias

#### PEDro scale

The results of the risk of bias assessment using the PEDro scale are shown in S1 Table. Absolute agreement was achieved in 183 out of 220 items (83.2%). A third assessor was consulted to judge two items (0.9%). The median PEDro score was 5 (range 3–8). Nine studies were considered as being of adequate quality according to the 6-point cut-off value. The following items were most commonly reported in the articles: random allocation (100%), between-group statistical comparison (95%), point measures and measures of variability (95%), and follow-up in more than 85% of subjects (80%). Blinding of the therapist was reported in none of the articles, blinding of the subject in one article (5%) and blinding of the assessor in seven articles (35%). A minority of articles reported that allocation was concealed (30%) and whether an intention-to-treat analysis was performed (35%). Half of the articles provided data on the similarity of the groups at baseline for most prognostic indicators and disease severity.

#### Cochrane Collaboration’s tool

The assessment of risk of bias using the Cochrane Collaboration’s tool is shown in S2 Table. Absolute agreement was achieved in 74 of 140 items (52.8%). For one item a third assessor was consulted to give the final judgment (0.7%). Two studies were considered to be of adequate quality, since the items random sequence generation, allocation concealment and blinding of outcome assessment could be rated as having low risk of bias. Reporting bias and attrition bias were considered as low in 90% and 65% of the articles respectively. With regard to selection bias, six articles (30%) reported adequate generation of a randomized sequence and two articles (10%) reported adequate allocation concealment. None of the articles were considered to have a low risk of bias on blinding of participants and personnel, while eight articles (40%) reported that outcome assessors were blinded to the allocated interventions. Eleven articles (55%) appeared to be free of other sources of bias. The most common reason to judge the other articles as having an unclear risk of bias at this domain was the insufficient reporting of the similarity between the study groups at baseline.

#### Therapeutic validity and risk bias

Table 3 presents the total scores on therapeutic validity and risk of bias. The scores on therapeutic validity of the physiotherapeutic exercise interventions seem not to correspond with the risk of bias scores. Three articles that did not meet any of the criteria of the CONTENT scale were also not considered to be of adequate quality according to the PEDro scale and the Cochrane Collaboration’s tool [39,42,45]. However, another article that did not fulfill any of the therapeutic validity criteria was considered to be of adequate quality according to both risk of bias assessment tools [36]. The only article that was considered to be of high therapeutic validity according to the cut-off value of six out of nine items was considered to be of adequate quality according to the Cochrane Collaboration’s tool, but not according to the PEDro scale [30].

**Table 3:** Overview of the total scores for therapeutic validity and risk of bias.

Study	Therapeutic validity	Risk of bias	
	CONTENT scale^a^	PEDro scale^a^	Cochrane Collaboration’s Tool^b^
*Strengthening exercise*			
Husby (2009)	5 (56%)	5 (50%)	No
Husby (2010)	5 (56%)	4 (40%)	No
Mikkelsen (2012)	5 (56%)	**7 (70%)**	No
Mikkelsen (2014)	**7 (78%)**	5 (50%)	**Yes**
Nankaku (2016)	2 (22%)	4 (40%)	No
Okoro (2016)	4 (44%)	**6 (60%)**	No
Suetta (2004)	2 (22%)	4 (40%)	No
*Aerobic exercise*			
Maire (2004)	2 (22%)	4 (40%)	No
Maire (2006)	4 (44%)	4 (40%)	No
*Functional exercise*			
Beaupre (2014)	0 (0%)	**7 (70%)**	**Yes**
Galea (2008)	2 (22%)	4 (40%)	No
Giaquinto (2010)	0 (0%)	3 (30%)	No
Heiberg (2012)	2 (22%)	**7 (70%)**	No
Heiberg (2016)	1 (11%)	**6 (60%)**	No
Johnsson (1988)	0 (0%)	3 (30%)	No
Monaghan (2016)	4 (44%)	**8 (80%)**	No
Umpierres (2014)	2 (22%)	**6 (60%)**	No
*Functional exercise and early full weight-bearing*			
Bodén (2004)	0 (0%)	**6 (60%)**	No
Monticone (2014)	2 (22%)	**7 (70%)**	No
Ström (2006)	0 (0%)	5 (50%)	No

Bold items indicate that the study is considered to be of adequate quality according to the assessment tool used.

^a^ Data are shown as total score (percentage of the maximal possible score).

^b^ Final judgment regarding adequate quality: yes (in case of low risk of bias for the items random sequence generation, allocation concealment and blinding of outcome assessment) or no.

When only taking into account the final judgments on risk of bias shown in Table 3, it can be seen that the PEDro scale and Cochrane Collaboration’s tool identify different sets of studies to be considered as having adequate quality. One study is rated as being of adequate quality according to both assessment tools [36]. The PEDro scale identifies eight additional articles, whereas the Cochrane Collaboration’s tool identifies one different additional article to be of adequate quality.

### Characteristics and effectiveness of physiotherapeutic exercise interventions

Table 1 shows that there is variation between the included studies regarding the characteristics of the physiotherapeutic exercise interventions, the control interventions and the type of outcome measures used. This variety also exists within the defined intervention categories (strengthening, aerobic and functional exercise). Three major characteristics of the included studies will be further described in this section.

#### Outcome measures

Three categories of outcome measures were predefined, based on the ICF model. Within these categories, different outcome measures and units of measurements were used. For joint and muscle function, hip muscle strength and hip ROM were the most evaluated outcomes. Hip muscle strength was measured in the following units of measurement: kilogram (kg) [27,28,45], Newton meter [29,33], Watts/kg body mass [30], Newton meter/kg body mass [30,31], Newton [32,42], pounds of force [36], pounds (lbs) [43] and Kendall’s criteria for manual muscle testing [46]. Hip ROM was displayed in degrees and was measured during active movement [40,41] or passive movement [31,42]. In one article it was not specified whether it was active or passive hip ROM [46].

In the functional performance category, the 6-minute walk test (6MWT) [32,36,38,40,41,43] and stair-climbing performance [30,32,33,38,40,41] were the most evaluated functional tests. In addition, maximal walking speed [29,33,36,42], ten-meter walking speed [30], one-legged stance [29], chair rise performance [30,32,33], the timed up-and-go test [31,32,38], the figure-of-eight test [40] and the index of muscle function [40] were used in the included articles to evaluate functional performance.

For self-reported outcomes, both disease-specific and generic questionnaires were used. The most commonly used disease-specific questionnaire was the Western Ontario and McMaster Universities Osteoarthritis Index (WOMAC): it was used in eight of the included articles to measure patient-reported pain, stiffness and/or physical function [29,34–36,38,39,43,44]. In addition, the Harris Hip Score [37,40], a tool that consists of both patient-reported outcomes (80% of the total score) and observations by a clinician (20% of the total score), and the Hip Disability and Osteoarthritis Outcome Score (HOOS) [30,40,41] were used. Lastly, one article reported the outcomes of the Japanese Orthopedic Association hip score (only the pain subcategory) [31]. The following generic questionnaires were used to assess health status: the 12-item or 36-item Short Form Health Survey (SF-12 or SF-36) [27,28,43,44,46], the Rand 36-item Health Survey (RAND-36) [36] and the EuroQol five dimensions questionnaire [29]. Patient-reported pain was assessed using the Visual Analogue Scale [29,43] and the numeric rating scale [44]. The Functional Independence Measure [44] was used as measurement for disability. In one article the method of pain assessment was not reported [42].

#### Control intervention

In eleven of the included articles, the CG followed a usual care protocol (also named standard or conventional rehabilitation). The other nine articles did not explicitly state whether the exercise program for the CG was usual care or if it was a protocol specifically developed for the trial. Nine of the included articles (45%) reported the frequency and intensity of the control intervention. The frequency varied from five to seven times a week. The intensity varied from 40 minutes to two hours or was reported as the number of repetitions of an exercise. Variety was also found in the setting of the control intervention (inpatient versus outpatient and land-based therapy versus a combination of land-based and aquatic therapy).

#### Length of follow-up

Length of follow-up in the included articles varied from fifteen days to five years postoperatively. Some articles reported results of both short-term (immediately after the intervention period) and long-term follow-up (after a period in which the intervention was no longer applied). Different results for the physiotherapeutic exercise interventions in time were found. In the article of Husby et al., muscle strength of the operated leg increased significantly more in the IG compared to the CG [27]. Directly after the four-week intervention period, leg press strength was 65.2% higher in the IG (76 kg vs 46 kg; p < 0.002) and abduction strength was 87.0% higher in the IG (43 kg vs 23 kg; p < 0.002). At one-year follow-up, no significant differences in muscle strength were found anymore when comparing the IG and CG (leg press strength: 95 kg vs 84 kg; abduction strength: 50 kg vs 38 kg). However, work efficiency was significantly higher in the IG compared to the CG (16.9% vs 13.0%, p = 0.047) [28]. Other articles found no between-group differences on hip abduction muscle strength up to twelve months postoperatively [36,45]. Heiberg et al. investigated the effects of a walking skill program [40], finding one year postoperatively a significant between-group difference in favor of the IG in covered distance on the 6MWT (535 m vs 483 m; p < 0.001) and stair-climbing performance (10 seconds vs 12 seconds; p = 0.05) [40]. These differences were no longer present five years postoperatively though: the covered distance on the 6MWT was 524 m in the IG compared to 530 m in the CG, and both groups obtained a mean score of 13 seconds on the stair-climbing test [41]. Beaupre et al. did not find significant differences between the IG and CG in the 6MWT and gait speed up to twelve months postoperatively [36]. For patient-reported outcomes, several articles found no between-group differences up to one year postoperatively, measured using the WOMAC [36], HOOS [30,40], SF-36 [27,28] and RAND-36 [36]. However, two articles found significantly lower WOMAC scores (indicating better health status or less disability) in favor of the IG at both short-term and long-term follow-up (up to six months [39] and twelve months [44]). Lastly, Maire et al. did not find a between-group difference in total WOMAC score two months postoperatively [34], whereas a significantly lower score was found for the IG compared to the CG at one year follow-up [35].

## Discussion

### Main findings

The aims of this systematic review were to determine the therapeutic validity and to assess the effectiveness of physiotherapeutic exercise interventions for joint and muscle function, functional performance and self-reported outcomes in patients following THA for OA. It was found that the therapeutic validity of physiotherapeutic exercise interventions was insufficient: only one out of the twenty articles included could be considered to be of high therapeutic validity. In addition, a minority of the studies was considered to be of adequate quality according to the two risk of bias assessment tools used. The therapeutic validity scores did not correspond with the risk of bias scores that were found. Due to the heterogeneity in characteristics of the physiotherapeutic exercise and control interventions, the length of follow-up and the outcome measures used in the trials, no clear evidence was found for the effectiveness of physiotherapeutic exercise following THA. Our hypothesis that studies of high therapeutic validity might be able to elicit positive effects of physiotherapeutic exercise following THA has therefore not been confirmed yet.

### Therapeutic validity

The insufficient therapeutic validity scores of the majority of the articles indicate that the potential effectiveness of the described physiotherapeutic exercise interventions is questionable. However, it cannot be stated whether the aspects of the CONTENT scale were not applied during the development of the intervention protocols or that these aspects were simply not reported in the articles. The lack of clear descriptions of the rationale and content of physiotherapeutic exercise interventions impedes an understanding of the focus and effect of such interventions. This issue has previously been reported by Bandholm & Kehlet, who state that a physiotherapeutic exercise intervention needs to be well described before the intervention can be implemented in clinical practice [9]. To this end, they give several suggestions for describing strengthening exercise (contraction types, time under tension, range of motion and relative load) and functional exercise (number of repetitions or number of minutes for a specific exercise). To increase insight into the potential effectiveness of rehabilitation methods following THA, it is therefore advised to make use of the CONTENT scale, in addition to a risk of bias assessment tool, when developing and reporting physiotherapeutic exercise interventions to be applied in RCTs.

Adequate patient selection was one of the least reported items of the CONTENT scale in the included articles. One point is awarded for this item if the goals of the therapeutic exercise match the participants’ problems (represented by bodily functions and structures, activities and participation levels). Adequate patient selection is an important aspect in clinical trials, as it is established that there might be a difference in response to exercise therapies between patients. Several factors have been found to influence this response, such as level of physical functioning [47], age, and amount of pain [48]. In this review only two articles addressed this adequacy of patient selection by including a specific population [30,44]. In the study of Mikkelsen et al. a preoperative HOOS ADL score ≤ 67 was defined as an inclusion criterion for participation in the study, yet while discussing their results the authors state that this cut-off value might still have been too high [30]. Monticone et al. only included patients who could not go home after discharge from the orthopedic unit due to comorbidities or insufficient home support [44]. Valid criteria for adequate patient selection should be defined and reported in clinical trials, otherwise drawing any conclusion on the evaluated intervention’s effect might be premature or even invalid. A suggestion could be to follow the criteria as proposed by the Osteoarthritis Research Society International (OARSI) in 2015 [49].

Three systematic reviews have previously been conducted in which the CONTENT scale was used to assess the therapeutic validity of physiotherapeutic exercise interventions in different patient populations [18–20]. Hoogeboom et al. assessed the therapeutic validity of preoperative exercise programs before primary total hip or knee replacement [18]. None of the twelve included studies met the criteria for having a high therapeutic validity [18]. Snoek et al. focused on aerobic exercise training in patients with heart disease, and found that three out of eight included studies met the requirements for therapeutic validity [19]. Lastly, Vooijs et al. investigated the effect of supervised physiotherapeutic exercise training in patients with chronic obstructive pulmonary disease [20]. Thirteen studies were included in this review, of which six were considered as having high therapeutic validity. In the studies of both Hoogeboom et al. and Vooijs et al., meta-regression analyses were performed to assess whether there was an association between therapeutic validity and the effects of the exercise interventions on functional recovery [18] or exercise capacity [20]. Hoogeboom et al. could not establish a significant association between therapeutic validity and the pooled effects for in-hospital functional recovery, short-term observed functional recovery and short-term self-reported functional recovery [18]. Vooijs et al. did not find an association between therapeutic validity and overall effect sizes for maximal exercise capacity and functional exercise capacity either [20]. These findings might indicate that the differences in therapeutic validity scores could be caused by insufficient reporting rather than true differences in the validity of the physiotherapeutic exercise interventions [20].

Since the CONTENT scale was developed in a Delphi study conducted over four rounds in which five experts in the field of therapeutic exercise participated [18], it can be assumed that its content validity (the degree to which the content of an instrument is an adequate reflection of the construct to be measured [50]) is sufficient. The developers of the CONTENT scale do state that the cut-off value of 6 points was chosen arbitrarily. They suggest that it needs to be determined whether this value can be considered as the appropriate threshold for reflecting high therapeutic validity. In the current review however, changing the threshold to 5 or 7 does not lead to a different conclusion (data not shown). The developers also acknowledge that the CONTENT scale might not only reflect the therapeutic validity of a physiotherapeutic exercise intervention but also the completeness of reporting such interventions. These aspects should be kept in mind when assessing the psychometric properties of the CONTENT scale. To further evaluate its validity and reliability, the CONTENT scale should be used in different fields in which physiotherapeutic exercise is frequently applied (e.g. orthopedic, rheumatologic, neurological, cardiopulmonary and cancer rehabilitation).

### Risk of bias

The majority of the included studies was judged as having potentially high risk of bias, using both the PEDro scale and the Cochrane Collaboration’s tool. This is in line with the most recent systematic review on this topic [23]. Nonetheless, both risk of bias assessment tools identified different sets of studies considered as being of adequate quality. This finding is in line with a meta-epidemiological study that compared both methods in assessing risk of bias of physiotherapeutic exercise trials [25]. The authors also concluded that the methods lead to different sets of trials considered to be of adequate quality, and thus to differences in combined effect sizes when meta-analyzing these trials [25]. In our experience, a notable difference between the two assessment tools can be seen when comparing the scores on the items random allocation and concealed allocation: the Cochrane Collaboration’s tool requires an explicit description of the method of random sequence generation, whereas the PEDro scale does not require a specification of the precise method of randomization, as long as the article states that allocation was random. Blinding of participants is often not possible in studies involving physiotherapeutic exercise interventions, so high risk of bias for this domain was present according to both risk of bias assessment tools. Since the other bias domains are less suitable to compare one-to-one between the two assessment tools, we are not able to specify what the exact origin of other differences in the final judgments might have been.

A review evaluating the Cochrane Collaboration’s tool found that it is often used in a non-recommended way [51]. Correct use of the tool could be improved by more guidance and training options. Difficulties in using the tool by researchers who have no experience with it are reflected in our review by the relatively low percentage of absolute agreement between assessors (52.8%), compared to the PEDro scale (83.2%) and the CONTENT scale (82.7%). Despite the challenges in using the assessment tool correctly, it is advised to use the Cochrane Collaboration’s tool rather than the PEDro scale to assess risk of bias in trials focusing on physiotherapeutic exercise [25]. With this tool, bias domains can receive different weights according to their relevance in a given context (as opposed to a summary score as reflected by the PEDro scale). Hence when using the Cochrane Collaboration’s tool to assess risk of bias in physiotherapeutic exercise trials the focus can lie on those bias domains which are most relevant to the objectives of the research at hand.

### Therapeutic validity and risk bias

In the current review, the levels of therapeutic validity did not correspond to the risk of bias scores. The only article considered as being of high therapeutic validity met the requirements of adequate quality according to the Cochrane Collaboration’s tool, but not according to the PEDro scale. The articles with insufficient therapeutic validity were mostly considered as being of insufficient quality according to the Cochrane Collaboration’s tool, but obtained varying scores according to the PEDro scale. Based on the current review, it is recommended that systematic reviews should not only assess risk of bias but also take the therapeutic validity of physiotherapeutic exercise interventions into account as both assess different aspects of the interventions.

### Characteristics and effectiveness of physiotherapeutic exercise interventions

The diversity in characteristics of physiotherapeutic exercise interventions, control interventions and outcome measures prevents a clear answer to the question of the extent to which physiotherapeutic exercise can improve joint and muscle function, functional performance and self-reported outcomes following THA. Even within the defined intervention categories (strengthening, aerobic and functional exercise) there is a wide variety regarding setting, supervision, duration, frequency, intensity, start and follow-up length of the interventions. Only one trial (which was presented in two articles) was identified that investigated the effects of aerobic exercise [34,35]. Generally small sample sizes were included, where the range was 7/7 (IG/CG) and 54/52 (IG/CG) as respectively the smallest and largest sample size.

Overall it can be concluded that more thorough research is essential. The OARSI clinical trials guidelines would be useful to follow. In these guidelines it is stated that thorough description of interventions is needed so that others can replicate it including algorithms for treatment selection/progression, dosage (intensity, frequency, duration), adherence strategies, home programs, training of treatment providers and treatment fidelity methods [51].

#### Outcome measures

Within the three different categories of outcome measures (joint and muscle function, functional performance and self-reported outcomes) there was a wide variety in used measurement instruments and reported units of measurement. In particular, hip muscle strength was expressed in different units of measurements across the articles. To conduct meta-analyses on muscle strength, uniformity of measurements is required. Differences in the scoring methods of the functional tests were found for functional performance. For example, chair rise performance assessed with the sit-to-stand test was rated as the maximal number of times patients were able to rise from a chair in 30 seconds in the study of Okoro et al. [32], while Suetta et al. counted the number of seconds in which patients were able to repeat rising from a chair five times [33]. The stair-climbing test was conducted in various ways, with differences in number and height of steps and in use of a handrail [30,32,33,38,40,41].

The earlier mentioned OARSI guidelines propose four categories of outcome measures: pain, patient-reported outcome measures, performance-based measures and patient global assessment. Regarding performance-based measures, a minimal core set is recommended to evaluate functional performance in hip and knee OA. This core set consists of three tests to evaluate performance of sit-to-stand (using the 30 seconds chair stand test), short walking distances (using the 4 x 10 meters fast-paced walk test) and stair negotiation tasks (using a timed stair task). The OARSI also developed a manual in which the protocols and psychometric properties of these tests are presented [52]. Moreover, authors should report all measures according to the International System of Units for comparability purposes.

Some of the articles in our review assessed both joint and muscle function and functional performance [29–33,36,40–43]. Results of these articles show that improvements in one category do not necessarily correspond with improvements in the other category and vice versa [30–33,40,43]. This is also described in the ICF model, which shows that an individual’s functioning can be defined at different levels and is the result of complex interactions between health condition and contextual factors [13]. Differences were also found in the results of self-reported outcomes compared to the other two categories of outcome measures. Previous research shows that there is a poor relationship between performance-based and self-reported measures of physical functioning in patients before and after THA, and that self-reported physical functioning is influenced by pain [53]. Therefore, to get a full impression of the effectiveness of physiotherapeutic exercise interventions, outcome measures of different categories, both performance-based and self-reported measures, should be included. With regard to self-reported questionnaires, it should be realized that pain could be a factor that negatively influences self-reported outcomes on other domains, such as physical functioning.

#### Control intervention

Due to limited information on the control interventions in the articles, there is limited evidence for the effectiveness of the physiotherapeutic exercise interventions. The majority of articles (55%) did not report on frequency and intensity of the control interventions. As a result, it is often not possible to assess whether a between-group difference is due to the exercise type or might be caused by a difference in exercise dose. To determine the effect of the exercise type, the interventions applied in both study groups should be as similar as possible in terms of the other characteristics (setting, supervision, frequency and intensity), and these characteristics should be clearly described.

The articles that did address the characteristics of the control intervention showed that there is variety in frequency and intensity between the different studies. The results also show that the “usual care” protocol may also vary considerably between countries and between clinical centers within countries. These differences in characteristics of control interventions between studies limit the ability to compare their results, so uniformity of control interventions across trials is desirable.

#### Length of follow-up

Both short-term and long-term results were reported in some articles, with length of long-term follow-up ranging from six months to five years. Long-term follow-up is required to assess whether any significant improvements are lasting, even after ending the intervention. For hip muscle strength and functional performance there seem to be no between-group differences in the long term after a significant short-term effect in favor of the IG [28,41]. However, several included articles that found significant improvement in favor of the IG directly after the intervention period did not assess the effects of the intervention in the long term. Future studies should therefore include long-term follow-up of participants to determine whether the effects of physiotherapeutic exercise interventions are lasting.

### Strengths and limitations

To our knowledge, this is the first systematic review to assess the therapeutic validity of physiotherapeutic exercise interventions following THA. In addition, we only included articles that focused on patients who underwent THA because of OA, and excluded articles that included patients with other conditions leading to THA (e.g. hip fracture, femoral head necrosis).

Several factors should be taken into account when interpreting the results of this systematic review. Although we searched five databases and included articles in three different languages, we might have missed articles relevant to our search. Furthermore, we did not contact the authors of the included articles for additional information when items from the therapeutic validity or risk of bias assessment tools were not reported in the article.

### Implications for future research

When designing and describing studies in which a physiotherapeutic exercise intervention is applied, both therapeutic validity and risk of bias should be taken into account. The CONTENT scale should be used to describe the various aspects of therapeutic validity to increase the transparency of interventions applied in clinical trials. This review demonstrates that the potential effectiveness of physiotherapeutic exercise following THA is still questionable, due to potentially inadequate patient selection, the wide variation in characteristics of physiotherapeutic exercise interventions, control interventions and outcome measures. To gain complete insight into the effectiveness of physiotherapeutic exercise interventions, future studies should include a spectrum of outcome measures for example as proposed by OARSI [51] and appropriate patient selection criteria. A clear description of the control intervention should also be provided in terms of exercise type, setting, supervision, frequency and intensity. Lastly, future studies should also include a long-term follow-up to assess whether any significant improvements after physiotherapeutic exercise interventions are lasting.

## Conclusion

Due to insufficient therapeutic validity of physiotherapeutic exercise interventions and potentially high risk of bias of the included studies, the effectiveness of physiotherapeutic exercise following THA for OA is unclear. Since the levels of therapeutic validity did not correspond to the risk of bias scores, both aspects should be taken into account when developing and reporting on protocols of clinical trials. In order to assess the effectiveness of physiotherapeutic exercise following THA, uniformity of characteristics of physiotherapeutic exercise interventions, control interventions, length of follow-up and outcome measures is necessary.

## Supporting information

S1 File PRISMA 2009 checklist. (DOC)

S2 File Search strategies for the different databases. (DOCX)

S3 File CONTENT scale to assess the therapeutic validity of therapeutic exercise programs. (DOCX)

S4 File List of excluded full-text articles. (DOCX)

S1 Table Results of the risk of bias assessment using the PEDro scale. (DOCX)

S2 Table Results of the risk of bias assessment using the Cochrane Collaboration’s tool. (DOCX)
