# Supplementary material for: The therapeutic validity and effectiveness of physiotherapeutic exercise following total hip arthroplasty for osteoarthritis: A systematic review
Source: PLoS One. 2018 Mar 16;13(3):e0194517. doi: 10.1371/journal.pone.0194517 (PMC5856403; doi:10.1371/journal.pone.0194517)
Supplement: S2 File — (DOCX) [file pone.0194517.s002.docx]

**S2 File. Search strategies for the different databases.**

Embase (468 hits)

('total hip prosthesis'/exp OR 'hip arthroplasty'/de OR 'hip prosthesis'/de OR (((hip OR hips) NEAR/6 (arthroplast* OR postarthroplast* OR replace* OR alloplast* OR prosthe*)) OR tha):ab,ti) AND ('physiotherapy'/exp OR 'exercise'/exp OR 'physical medicine'/de OR physiotherapist/de OR 'kinesiotherapy'/exp OR 'rehabilitation'/de OR 'rehabilitation':lnk OR 'rehabilitation medicine'/de OR 'functional training'/de OR mobilization/de OR (((physio OR physical OR manual) NEAR/3 (therap* OR treat* OR medicine)) OR ((muscle* OR quadricep* OR abductor* OR gluteal*) NEAR/3 (train* OR strengthen*)) OR ((strength* OR function*) NEAR/3 train*) OR physiotherap* OR mobilisat* OR mobilizat* OR exercis* OR kinesiotherap* OR kinesitherap* OR rehabilitat*):ab,ti) AND ((random* OR factorial* OR crossover* OR (cross NEXT/1 over*) OR placebo* OR ((doubl* OR singl*) NEXT/1 blind*) OR assign* OR allocat* OR volunteer*):ab,ti OR 'crossover procedure'/de OR 'double-blind procedure'/de OR 'randomized controlled trial'/de OR 'single-blind procedure'/de) NOT ([animals]/lim NOT [humans]/lim) NOT ([Conference Abstract]/lim OR [Letter]/lim OR [Note]/lim OR [Editorial]/lim)

MEDLINE (731 hits)

("Arthroplasty, Replacement, Hip"/ OR "Hip Prosthesis"/ OR (((hip OR hips) ADJ6 (arthroplast* OR postarthroplast* OR replace* OR alloplast* OR prosthe*)) OR tha).ab,ti.) AND ("Physical Therapy Modalities"/ OR exp "Exercise Movement Techniques"/ OR exp "Exercise Therapy"/ OR exp "Exercise"/ OR "Physical and Rehabilitation Medicine"/ OR Physical Therapists/ OR "Physical Therapy Specialty"/ OR "Rehabilitation"/ OR "rehabilitation".xs. OR (((physio OR physical OR manual) ADJ3 (therap* OR treat* OR medicine)) OR ((muscle* OR quadricep* OR abductor* OR gluteal*) ADJ3 (train* OR strengthen*)) OR ((strength* OR function*) ADJ3 train*) OR physiotherap* OR mobilisat* OR mobilizat* OR exercis* OR kinesiotherap* OR kinesitherap* OR rehabilitat*).ab,ti.) AND (exp Clinical Trial/ OR randomized.ab,ti. OR placebo.ab,ti. OR randomly.ab,ti. OR trial.ab,ti. OR groups.ab,ti. NOT (Animals/ NOT Humans/)) NOT (exp animals/ NOT humans/) NOT (letter OR news OR comment OR editorial OR congresses OR abstracts).pt.

The Cochrane Library (397 hits)

((((hip OR hips) NEAR/6 (arthroplast* OR postarthroplast* OR replace* OR alloplast* OR prosthe*)) OR tha):ab,ti) AND ((((physio OR physical OR manual) NEAR/3 (therap* OR treat* OR medicine)) OR ((muscle* OR quadricep* OR abductor* OR gluteal*) NEAR/3 (train* OR strengthen*)) OR ((strength* OR function*) NEAR/3 train*) OR physiotherap* OR mobilisat* OR mobilizat* OR exercis* OR kinesiotherap* OR kinesitherap* OR rehabilitat*):ab,ti)

CINAHL (303 hits) and AMED (68 hits)

(MH "Arthroplasty, Replacement, Hip" OR TX (((hip OR hips) N6 (arthroplast* OR postarthroplast* OR replace* OR alloplast* OR prosthe*)) OR tha)) AND ((MH "Physical Therapy+") OR (MH "Therapeutic Exercise+") OR (MH "Gait Training+") OR (MH "Functional Training") OR (MH "Home Physical Therapy") OR (MH "Joint Mobilization") OR (MH "Exercise+") OR (MH "Physical Therapists") OR (MH "Rehabilitation+") OR TX (((physio OR physical OR manual) N3 (therap* OR treat* OR medicine)) OR ((muscle* OR quadricep* OR abductor* OR gluteal*) N3 (train* OR strengthen*)) OR ((strength* OR function*) N3 train*) OR physiotherap* OR mobilisat* OR mobilizat* OR exercis* OR kinesiotherap* OR kinesitherap* OR rehabilitat*)) AND ((MH "Randomized Controlled Trials" OR TI (random* OR trial) OR AB (random*))
