# Supplementary material for: The therapeutic validity and effectiveness of physiotherapeutic exercise following total hip arthroplasty for osteoarthritis: A systematic review
Source: PLoS One. 2018 Mar 16;13(3):e0194517. doi: 10.1371/journal.pone.0194517 (PMC5856403; doi:10.1371/journal.pone.0194517)
Supplement: S3 File — (DOCX) [file pone.0194517.s003.docx]

**S3 File. CONTENT scale to assess the therapeutic validity of therapeutic exercise programs [16].**

| **Items** | | **Judgement** | |
| --- | --- | --- | --- |
| *A. Patient eligibility* | |  |  |
| 1. | Was the patient selection described? | Yes | No |
|  | To score “yes”, patient selection should be described and participants should be screened for contraindications (for instance, using red and yellow flags) (this must be explicitly mentioned in the manuscript; otherwise “no”). |  |  |
| 2. | Was the patient selection adequate? | Yes | No |
|  | This item can be scored as “yes” if:  - the goals of the therapeutic exercise match the participants’ problems (for instance, if the goal of the therapeutic exercise is to improve a patients’ functional status, then only patients with deprived functional status should be included). In this case participants’ problems represent bodily functions and structures, activities and participation levels, see the ‘International Classification of Functioning, Disability and Health (ICF); and  - the selection criteria match the majority of potential participants. Ergo, the therapeutic exercise should not be evaluated in a population that -in clinical practice- is nearly non-existent. |  |  |
| *B. Competences and setting* | |  |  |
| 3. | Were eligibility criteria for therapist and setting determined and adequate? | Yes | No |
|  | The questions to be answered here are:  - Are the goals and content of the therapeutic exercise matched to the therapist’s competences and skills?  - Are the goals and content of the therapeutic exercise matched to the location or setting where the therapeutic exercise takes place?  If no eligibility criteria are described, this item should be scored as “no”. |  |  |
| *C. Rationale* | |  |  |
| 4. | Was the therapeutic exercise based on a-priori aims and intentions? | Yes | No |
|  | Did the authors describe a-priori aims, intentions and hypotheses about the therapeutic exercise on theoretically driven and/or argued choices? If this question can be answered with “yes”, this item is scored as “yes”. |  |  |
| 5. | Was the rationale for the content and intensity of the therapeutic exercise described and plausible? | Yes | No |
|  | Did the authors describe why they believed the content (e.g. resistance exercise training, aerobic exercise training, flexibility training, etc.) and intensity (e.g. moderate/vigorous intensity, length of exercise, etc.) of the studies intervention was likely to achieve their treatment goals? |  |  |
| *D. Content* | |  |  |
| 6. | Was the intensity of the therapeutic exercise described? | Yes | No |
|  | This item can be scored as “yes” if:  - the content of the therapeutic exercise is described in specific terms (i.e. duration, frequency and intensity of exercise sessions (e.g. 80% VO_2max_, level of exertion (RPE), repetition maximum, etc.) and the total duration of the therapeutic exercise);  - the intensity of the therapeutic exercise was selected and adjusted of theoretically driven and/or argued choices; and  - the content of the therapeutic intervention is suitable for the majority of participants. |  |  |
| 7. | Was the therapeutic exercise monitored and adjusted when considered necessary? | Yes | No |
|  | This item can be scored as “yes” if:  1. the regular and structured monitoring of therapy progression allows the therapist to:  - strive for optimal exercise intensity;  - adjust the intervention in case of therapy failure on an individual level; and  - identify and monitor adverse effects.  2. the outcome measures match the therapy goals. |  |  |
| 8. | Was the therapeutic exercise personalised and contextualised to the individual participants? | Yes | No |
|  | The goals and content of the therapeutic exercise should not only match the patients’ bodily functions and structures, activities and participation levels, but also their personal and environmental factors (see ICF). This item can be scored as “yes” if the therapeutic exercise accounts for relevant personal (e.g. motivation, coping, ethnicity, etc.) and environmental (e.g. logistics, support family/friends, products and technology, etc.) factors for each of the included participants. |  |  |
| *E. Adherence* | |  |  |
| 9. | Was adherence to the therapeutic exercise determined and acceptable? | Yes | No |
|  | For adherence to be properly described and acceptable, adherence should be described in such a way that it allows the reader to understand whether the actual executed therapeutic exercise differed from the planned therapeutic exercise (i.e. data should be provided on the achieved intensity, for example number of sessions attended, achieved exercise intensity, number of exercises, etc.). Moreover, adherence should be quantitatively known, allowing it to be controlled for in the analysis. |  |  |
