# Supplementary material for: The therapeutic validity and effectiveness of physiotherapeutic exercise following total hip arthroplasty for osteoarthritis: A systematic review
Source: PLoS One. 2018 Mar 16;13(3):e0194517. doi: 10.1371/journal.pone.0194517 (PMC5856403; doi:10.1371/journal.pone.0194517)
Supplement: S4 File — (DOCX) [file pone.0194517.s004.docx]

**S4 File. List of excluded full-text articles.**

| Exclusion criterion 1: no separate results for total hip arthroplasty for osteoarthritis |
| --- |
| Bulthuis Y, Drossaers-Bakker KW, Taal E, Rasker J, Oostveen J, van't Pad Bosch P, Oosterveld F, van de Laar M. Arthritis patients show long-term benefits from 3 weeks intensive exercise training directly following hospital discharge. Rheumatology (UK) 2007;46(11):1712-7. |
| Hauer K, Specht N, Schuler M, B��rtsch P, Oster P. Intensive physical training in geriatric patients after severe falls and hip surgery. Age Ageing 2002;31(1):49-57. |
| Hesse S, Werner C, Seibel H, Von Frankenberg S, Kappel E-, Kirker S, K��ding M. Treadmill training with partial body-weight support after total hip arthroplasty: A randomized controlled trial. Arch Phys Med Rehabil 2003;84(12):1767-73. |
| Jogi P, Overend TJ, Spaulding SJ, Zecevic A, Kramer JF. Effectiveness of balance exercises in the acute post-operative phase following total hip and knee arthroplasty: A randomized clinical trial. SAGE Open Med 2015;3. |
| Jogi P, Zecevic A, Overend TJ, Spaulding SJ, Kramer JF. Force-plate analyses of balance following a balance exercise program during acute post-operative phase in individuals with total hip and knee arthroplasty: A randomized clinical trial. SAGE Open Med 2016;4. |
| Kishida Y, Sugano N, Sakai T, Nishii T, Haraguchi K, Ohzono K, Yoshikawa H. Full weight-bearing after cementless total hip arthroplasty. Int Orthop 2001;25(1):25-8. |
| Liebs TR, Herzberg W, Rther W, Haasters J, Russlies M, Hassenpflug J. Multicenter randomized controlled trial comparing early versus late aquatic therapy after total hip or knee arthroplasty. Arch Phys Med Rehabil 2012;93(2):192-9. |
| Liebs TR, Herzberg W, Ruther W, Haasters J, Russlies M, Hassenpflug J. Ergometer cycling after hip or knee replacement surgery: A randomized controlled trial. J Bone Joint Surg Am 2010 Apr;92(4):814-22. |
| Mahomed NN, Davis AM, Hawker G, Badley E, Davey JR, Syed KA, Coyte PC, Gandhi R, Wright JG. Inpatient compared with home-based rehabilitation following primary unilateral total hip or knee replacement: A randomized controlled trial. J Bone Jt Surg Ser A 2008;90(8):1673-80. |
| Scherak O, Kolarz G, Wottawa A, Maager M, el Shohoumi M. [Comparison between early and late inpatient rehabilitation measures after implantation of total hip endoprostheses]. Rehabilitation (Stuttg) 1998 Aug;37(3):123-7. |
| Steinhilber B, Haupt G, Miller R, Boeer J, Grau S, Janssen P, Krauss I. Feasibility and efficacy of an 8-week progressive home-based strengthening exercise program in patients with osteoarthritis of the hip and/or total hip joint replacement: A preliminary trial. Clin Rheumatol 2012;31(3):511-9. |
| Werner C, Kappel EM, Sonntag D, Bardeleben A, Kading M, Hesse S. Treadmill therapy with partial body weight support after total hip arthroplasty. german. Physikalische Medizin Rehabilitationsmedizin Kurortmedizin 2004;14(3):140-5. |

| Exclusion criterion 2: conference abstract or poster |
| --- |
| Elibol N, Unver B, Karatosun V. Effectiveness of balance exercises on falling risk in the acute post-operative period following total hip arthroplasty-A pilot study. HIP International.Conference: 12th Congress of the European Hip Society, EHS 2016.Germany.Conference Start: 20160906.Conference End: 20160909 2017;26:S19. |
| Heiberg KE, Figved W. Exercise, recovery of physical functioning, and prediction of physical activity after total hip arthroplasty. 5-year follow-up of a rct. Ann Rheum Dis 2015;74:1318-9. |
| Marcu IR, Patru S, Bighea A, Popescu RS, Bumbea AM. Effect of hydrotherapy vs. conventional land-based exercise in patients with hiparthroplasty for osteoarthritis. Osteoporosis Int 2014;25:S421. |
| Monaghan B, Blake C, Hing W, Cusack T. Functional exercise after total hip replacement (feather). Physiotherapy (United Kingdom) 2015;101:eS1024-5. |
| Tsukagoshi R, Tateuchi H, Fukumoto Y, Okumura H, Ichihashi N. Stepping exercise improves muscle strength in the early postoperative phase after total hip arthroplasty: A clinical controlled trial. Physiotherapy (United Kingdom) 2011;97:eS1258. |

| Exclusion criterion 3: language |
| --- |
| Wilk M, Franczuk B. Evaluating changes in the range of movement in the hip joint in patients with degenerative changes, before and after total hip replacement. Ortop Traumatol Rehabil 2004 Jun;6(3):342-9. |
| Yang M, Wang H-, Qin D-, Guo X-. Gluteus maximus and gluteus medius exercise following elder total hip arthroplasty. Journal of Clinical Rehabilitative Tissue Engineering Research 2011;15(17):3202-5. |
| Zak M, Skalska A. Walking speed in elderly patients undergoing rehabilitation after hip replacement. Ortop Traumatol Rehabil 2005 Oct;7(5):543-8. |

| Exclusion criterion 4: no randomized controlled trial |
| --- |
| Kolarz G, Maager M, Scherak O, eL Shohoumi M, Wottawa A. Rehabilitation after total hip replacement. Int J Rehabil Res 1995 Sep;18(3):266-9. |
| Petershagen A, Bendorf R. Ist die huftgelenkbeweglichkeit nach huftendoprothetik ein gradmesser fur den erfolg einer anschlussheilbehandlung? KRANKEN GYMNASTIK 1996;48(8):1188. |
| Scherak O, Kolarz G, Wottawa A, Maager M, el Shohoumi M. [Effect of inpatient rehabilitation measures on patients with total hip endoprostheses--evaluation 15 months after operation]. Acta Med Austriaca 1996;23(4):142-5. |

| Exclusion criterion 5: reason for total hip arthroplasty unknown |
| --- |
| Smith TO, Mann CJV, Clark A, Donell ST. Bed exercises following total hip replacement: A randomised controlled trial. Physiotherapy 2008;94(4):286-91. |
| Smith TO, Mann CJV, Clark A, Donell ST. Bed exercises following total hip replacement: 1 year follow-up of a single-blinded, randomised controlled trial. HIP Int 2009;19(3):268-73. |

| Exclusion criterion 6: no relevant outcome measures |
| --- |
| Suetta C, Andersen JL, Dalgas U, Berget J, Koskinen S, Aagaard P, Magnusson SP, Kjaer M. Resistance training induces qualitative changes in muscle morphology, muscle architecture, and muscle function in elderly postoperative patients. J Appl Physiol 2008;105(1):180 |

| Exclusion criterion 7: no baseline measurement |
| --- |
| Grange CC, Maire J, Groslambert A, Tordi N, Dugue B, Pernin J-, Rouillon J-. Perceived exertion and rehabilitation with arm crank in elderly patients after total hip arthroplasty: A preliminary study. J Rehabil Res Dev 2004;41(4):611-9. |

| Exclusion criterion 8: bilateral total hip arthroplasty included |
| --- |
| Morishima Y, Mizushima T, Yamauchi K, Morikawa M, Masuki S, Nose H. Effects of home-based interval walking training on thigh muscle strength and aerobic capacity in female total hip arthroplasty patients: A randomized, controlled pilot study. PLoS ONE 2014;9(9). |

| Exclusion criterion 9: time since total hip arthroplasty longer than six months |
| --- |
| Patterson AJ, Murphy NM, Nugent AM, Finlay OE, Nicholls DP, Boreham CA, Steele I, Henderson SA, Beringer TR. The effect of minimal exercise on fitness in elderly women after hip surgery. Ulster Med J 1995;64(2):118 |
