# Supplementary material for: The therapeutic validity and effectiveness of physiotherapeutic exercise following total hip arthroplasty for osteoarthritis: A systematic review
Source: PLoS One. 2018 Mar 16;13(3):e0194517. doi: 10.1371/journal.pone.0194517 (PMC5856403; doi:10.1371/journal.pone.0194517)
Supplement: S1 Table — (DOCX) [file pone.0194517.s005.docx]

**S1 Table. Results of the risk of bias assessment using the PEDro scale.**

| Study | Random allocation | Concealed allocation | Groups similar at baseline | Subject blinding | Therapist blinding | Assessor blinding | Follow-up > 85% | Intention-to-treat analysis | Between-group statistical comparison | Point measures and measures of variability | Total score |
| --- | --- | --- | --- | --- | --- | --- | --- | --- | --- | --- | --- |
| *Strengthening exercise* | | | | | | | | | | | |
| Husby (2009) | Yes | No | Yes | No | No | No | Yes | No | Yes | Yes | 5 |
| Husby (2010) | Yes | No | No | No | No | No | Yes | No | Yes | Yes | 4 |
| Mikkelsen (2012) | Yes | Yes | No | No | No | Yes | Yes | Yes | Yes | Yes | 7 |
| Mikkelsen (2014) | Yes | Yes | No | No | No | No | No | Yes | Yes | Yes | 5 |
| Nankaku (2016) | Yes | No | Yes | No | No | No | Yes | No | No | Yes | 4 |
| Okoro (2016) | Yes | No | Yes | No | No | Yes | No | Yes | Yes | Yes | 6 |
| Suetta (2004) | Yes | No | Yes | No | No | No | No | No | Yes | Yes | 4 |
| *Aerobic exercise* | | | | | | | | | | | |
| Maire (2004) | Yes | No | No | No | No | No | Yes | No | Yes | Yes | 4 |
| Maire (2006) | Yes | No | No | No | No | No | Yes | No | Yes | Yes | 4 |
| *Functional exercise* | | | | | | | | | | | |
| Beaupre (2014) | Yes | Yes | Yes | No | No | Yes | Yes | No | Yes | Yes | 7 |
| Galea (2008) | Yes | No | No | No | No | No | Yes | No | Yes | Yes | 4 |
| Giaquinto (2010) | Yes | No | No | No | No | No | Yes | No | Yes | No | 3 |
| Heiberg (2012) | Yes | No | Yes | No | No | Yes | Yes | Yes | Yes | Yes | 7 |
| Heiberg (2016) | Yes | No | No | No | No | Yes | Yes | Yes | Yes | Yes | 6 |
| Johnsson (1988) | Yes | No | No | No | No | No | No | No | Yes | Yes | 3 |
| Monaghan (2016) | Yes | Yes | Yes | No | No | Yes | Yes | Yes | Yes | Yes | 8 |
| Umpierres (2014) | Yes | No | No | Yes | No | Yes | Yes | No | Yes | Yes | 6 |
| *Functional exercise and early full weight-bearing* | | | | | | | | | | | |
| Bodén (2004) | Yes | Yes | Yes | No | No | No | Yes | No | Yes | Yes | 6 |
| Monticone (2014) | Yes | Yes | Yes | No | No | No | Yes | Yes | Yes | Yes | 7 |
| Ström (2006) | Yes | No | Yes | No | No | No | Yes | No | Yes | Yes | 5 |
| Total score | 20 (100%) | 6 (30%) | 10 (50%) | 1 (5%) | 0 (0%) | 7 (35%) | 16 (80%) | 7 (35%) | 19 (95%) | 19 (95%) |  |
