# Supplementary material for: The therapeutic validity and effectiveness of physiotherapeutic exercise following total hip arthroplasty for osteoarthritis: A systematic review
Source: PLoS One. 2018 Mar 16;13(3):e0194517. doi: 10.1371/journal.pone.0194517 (PMC5856403; doi:10.1371/journal.pone.0194517)
Supplement: S2 Table — (DOCX) [file pone.0194517.s006.docx]

**S2 Table. Results of the risk of bias assessment using the Cochrane Collaboration’s tool.**

|  | Selection bias | |  | Performance bias |  | Detection bias |  | Attrition bias |  | Reporting bias |  | Other bias |  |  |
| --- | --- | --- | --- | --- | --- | --- | --- | --- | --- | --- | --- | --- | --- | --- |
| Study | Random sequence generation | Allocation concealment |  | Blinding of participants and personnel |  | Blinding of outcome assessment |  | Incomplete outcome data |  | Selective reporting |  | Other sources of bias |  | Adequate quality^a^ |
| *Strengthening exercise* | | | | | | | | | | | | | | |
| Husby (2009) | Low | Unclear |  | High |  | Unclear |  | Low |  | Low |  | Low |  | No |
| Husby (2010) | Low | Unclear |  | High |  | Unclear |  | High |  | Low |  | Unclear |  | No |
| Mikkelsen (2012) | Unclear | Unclear |  | High |  | Low |  | Low |  | Low |  | Unclear |  | No |
| Mikkelsen (2014) | Low | Low |  | High |  | Low |  | Low |  | Unclear |  | Unclear |  | Yes |
| Nankaku (2016) | Unclear | Unclear |  | High |  | Unclear |  | Low |  | Low |  | Low |  | No |
| Okoro (2016) | Unclear | Unclear |  | High |  | Low |  | High |  | Low |  | Low |  | No |
| Suetta (2004) | Low | Unclear |  | High |  | Unclear |  | Unclear |  | Low |  | Low |  | No |
| *Aerobic exercise* | | | | | | | | | | | | | | |
| Maire (2004) | Unclear | Unclear |  | High |  | Unclear |  | Low |  | Low |  | Unclear |  | No |
| Maire (2006) | Unclear | Unclear |  | High |  | Unclear |  | Unclear |  | Low |  | Unclear |  | No |
| *Functional exercise* | | | | | | | | | | | | | | |
| Beaupre (2014) | Low | Low |  | High |  | Low |  | Low |  | Low |  | Low |  | Yes |
| Galea (2008) | Unclear | Unclear |  | High |  | Unclear |  | Unclear |  | Low |  | Unclear |  | No |
| Giaquinto (2010) | Unclear | Unclear |  | High |  | High |  | Unclear |  | Low |  | Unclear |  | No |
| Heiberg (2012) | Unclear | Unclear |  | High |  | Low |  | Low |  | Low |  | Low |  | No |
| Heiberg (2016) | Unclear | Unclear |  | High |  | Low |  | Low |  | Low |  | Low |  | No |
| Johnsson (1988) | Unclear | Unclear |  | High |  | Unclear |  | Unclear |  | Low |  | Unclear |  | No |
| Monaghan (2016) | Low | Unclear |  | High |  | Low |  | Low |  | Unclear |  | Low |  | No |
| Umpierres (2014) | High | Unclear |  | Unclear |  | Low |  | Low |  | Low |  | Unclear |  | No |
| *Functional exercise and early full weight-bearing* | | | | | | | | | | | | | | |
| Bodén (2004) | Unclear | Unclear |  | High |  | Unclear |  | Low |  | Low |  | Low |  | No |
| Monticone (2014) | Unclear | Unclear |  | High |  | High |  | Low |  | Low |  | Low |  | No |
| Ström (2006) | Unclear | Unclear |  | High |  | Unclear |  | Low |  | Low |  | Low |  | No |
| Total items rated low’ | 6 (30%) | 2 (10%) |  | 0 (0%) |  | 8 (40%) |  | 13 (65%) |  | 18 (90%) |  | 11 (55%) |  |  |

^a^ Studies were considered to be of adequate quality when the items random sequence generation, allocation concealment and blinding of outcome assessment were rated as low risk of bias.
